# Hypoxia-Inducible Factor 1α in Type 2 Diabetes Mellitus: Mechanisms and Therapeutic Perspectives

**DOI:** 10.3390/biology15151241

**Published:** 2026-07-28

**Authors:** Junjing Jia, Qianying Lu, Yingyi Zhang, Yanmei Zhao

**Affiliations:** 1 School of Disaster and Emergency Medicine, Tianjin University, Tianjin 300072, China; 2024246222@tju.edu.cn (J.J.); qianying.lu@tju.edu.cn (Q.L.); 2 Tianjin Key Laboratory of Disaster Medicine Technology, Tianjin 300072, China

**Keywords:** diabetic complications, hypoxia-inducible factor 1α, metabolic reprogramming, molecular targets, T2DM

## Abstract

Type 2 diabetes mellitus (T2DM) is a global epidemic driven by chronic hyperglycemia, leading to severe microvascular and macrovascular complications. Hypoxia is a critical pathogenic factor, and hypoxia-inducible factor 1α (HIF-1α) serves as a central transcriptional regulator of cellular adaptation to low oxygen. This review examines the dual roles of HIF-1α in T2DM and its complications, including diabetic kidney disease, retinopathy, obesity-related insulin resistance, and impaired wound healing. HIF-1α exerts protective effects by enhancing glycolysis, reducing oxidative stress, and promoting angiogenesis, but it can also be detrimental by driving pathological neovascularization, fibrosis, and chronic inflammation. Its functional outcomes depend on tissue type, disease stage, and metabolic context, posing therapeutic challenges. Emerging pharmacologic strategies aim to fine-tune HIF-1α activity. A deeper understanding of the spatiotemporal regulation of HIF-1α signaling may inform more targeted therapeutic strategies, though considerable preclinical and clinical challenges remain.

## Simple Summary

Type 2 diabetes is a widespread disease that affects hundreds of millions of people worldwide. It occurs when the body cannot properly use or produce insulin, leading to high blood sugar levels that can damage vital organs such as the kidneys, eyes, and blood vessels. This review focuses on hypoxia-inducible factor 1α (HIF-1α), an oxygen-sensitive regulatory subunit that helps cells adapt to low oxygen. In diabetes, HIF-1α can function as either a protector or a driver of disease, depending on the cell type, tissue, and disease stage. It may support metabolic adaptation and tissue repair, but persistent or excessive activation can promote pathological angiogenesis, fibrosis, inflammation, and insulin resistance. Understanding this context-dependent role may help guide the development of more selective therapeutic strategies, although current evidence is derived mainly from preclinical studies and clinical validation remains limited.

## 1. Introduction

Diabetes mellitus (DM) is a major component of the metabolic syndrome, which is defined as the coexistence of elevated blood pressure, insulin resistance, obesity and dyslipidemia, in particular hypertriglyceridemia and a reduced ratio of high-density lipoprotein (HDL) to low-density lipoprotein (LDL) [1]. DM is characterized by chronic hyperglycemia resulting from impaired insulin secretion, insulin resistance, or both. Prolonged hyperglycemia drives systemic damage and dysfunction in critical organs, including the kidneys, retina, and cardiovascular system [2].

In 2021, diabetes affected approximately 537 million adults worldwide, with the number projected to increase to 643 million by 2030 and 783 million by 2045. Type 2 diabetes mellitus (T2DM) accounts for more than 90% of all diabetes cases [3,4]. T2DM arises from insulin resistance coupled with progressive pancreatic β-cell dysfunction, leading to chronic hyperglycemia and systemic metabolic stress. These derangements promote both microvascular complications (diabetic nephropathy, retinopathy, and neuropathy) and macrovascular diseases (cardiovascular disease and stroke), which remain leading causes of disability and mortality [5]. The escalating global burden underscores the urgent need for improved prevention and treatment strategies.

Hypoxia, defined as oxygen levels below physiological requirements, has recently been identified as a central factor in the pathogenesis of diabetes. Hypoxic conditions have been reported in the retina, kidney, pancreatic islets, adipose tissue, and skin of diabetic patients, indicating that insufficient oxygen supply is directly involved in disease progression and complications [6].

Oxygen is essential for aerobic metabolism and cellular energy production through mitochondrial oxidative phosphorylation. Disruption of oxygen homeostasis induces a hypoxic state, which reduces ATP generation, disturbs the redox balance, and activates a cascade of pathological events. At the molecular level, hypoxia stabilizes hypoxia inducible factors (HIFs), which initiate transcriptional programs that promote cellular adaptation but can also increase oxidative stress through mitochondrial electron transport chain dysfunction [7,8]. During this process, the HIF-α subunit (primarily HIF-1α) is stabilized and translocated to the cell nucleus, where it subsequently binds to HIF-1β to form a heterodimer; this heterodimer initiates downstream transcriptional programs by recognizing and binding to the hypoxia response element (HRE) within the regulatory elements of target genes.

HIF-1α is a central regulator of oxygen homeostasis and metabolic reprogramming. In diabetes, hyperglycemia has been shown to suppress HIF-1α activity in a tissue-specific manner, leading to impaired adaptation to hypoxia and aggravation of cellular injury [6]. Nevertheless, this suppression is not uniform across all cell types; in certain contexts, such as glomerular mesangial cells and adipose tissue of obese individuals, hyperglycemia or hypoxia can instead stabilize HIF-1α [9,10], underscoring that the regulation of HIF-1α is highly dependent on cell type and microenvironment. HIF-1α links hypoxic signaling with the pathophysiology of T2DM and its complications, which makes it an important therapeutic target.

Literature Search Strategy. A targeted narrative literature search was conducted in PubMed and Web of Science using combinations of the terms “HIF-1α”, “HIF-1”, “HIF-2α”, “type 2 diabetes”, “insulin resistance”, “diabetic kidney disease”, “diabetic retinopathy”, “diabetic wound healing”, and “obesity”. English-language articles published from 2000 to 2026 were considered, with emphasis on original mechanistic studies, clinical evidence, and recent high-quality reviews. Additional studies were identified by screening the reference lists of relevant articles.

## 2. Pathophysiology of Diabetes Mellitus

DM encompasses a group of metabolic disorders defined by chronic hyperglycemia, which over time leads to progressive dysfunction and damage in multiple organs, including the eyes, kidneys, nervous system, and cardiovascular tissues [11,12]. DM is broadly classified into type 1 (T1DM) and type 2 (T2DM), with the latter accounting for the vast majority of cases [13]. T2DM has become a global epidemic, with its prevalence more than doubling over the past two decades, and Asia emerging as a major epicentre of this rapidly growing burden [14,15]. The disease is characterized by insulin resistance, dysregulated glucose metabolism, and progressive β-cell failure, which manifest as reduced cellular responsiveness to insulin, impaired glucose uptake, and inadequate compensatory insulin secretion [16]. These metabolic derangements frequently lead to severe complications, including visual impairment, cardiovascular disease, and peripheral vascular damage [17,18]. Accumulating evidence highlights obesity-associated chronic low-grade inflammation as a key contributor to T2DM pathogenesis [4]. Given its high prevalence and strong links to modifiable lifestyle factors, T2DM remains a major research focus. This review therefore concentrates on the pathogenesis and complications of T2DM, as well as the regulatory role of HIF-1α in these processes.

Pancreatic β-cells require substantial oxygen for mitochondrial respiration to meet the energy demands of insulin synthesis and secretion. Under metabolic overload, heightened oxygen utilization induces intracellular hypoxia [19]. Experimental studies reveal that β-cells exposed to elevated glucose levels in vitro adopt a hypoxic state, stabilizing HIF-1α [20,21]. HIF-1α upregulates the expression of hypoxia-responsive genes, including vascular endothelial growth factor (VEGF) to mediate angiogenesis, erythropoietin (EPO) to stimulate erythropoiesis, and glycolytic enzymes that facilitate glucose metabolism [21]. This metabolic shift prioritizes glycolysis over mitochondrial ATP generation, a survival mechanism under oxygen scarcity [21]. Genetic mouse models with β-cell-specific deletion of HIF-1α or HIF-1β exhibit glucose intolerance resulting from impaired insulin secretion, independent of islet cell loss, which underscores the essential contribution of hypoxic signaling to β-cell functional regulation [19,22]. Persistent failure to mount adequate hypoxic responses may therefore accelerate β-cell deterioration [19]. As a result, timely activation of mechanisms adapted to hypoxia is required for regulation, and HIF-1α is an important factor in the corresponding hypoxic response. Collectively, these findings suggest that HIF-1α helps β-cells adapt to metabolic stress. Under hypoxic conditions, its transient activation may facilitate glycolytic compensation and insulin secretion, whilst sustained or excessive activation may impair mitochondrial metabolism and β-cell function. Consequently, both insufficient and excessive HIF-1α activity may have adverse effects, highlighting the double-edged nature of HIF-1α in the pathogenesis of diabetes.

## 3. Family of Hypoxia-Inducible Factors (HIFs)

The HIFs family members are heterodimeric proteins composed of oxygen-sensitive α-subunits (HIF-1α, HIF-2α, HIF-3α) and a constitutively expressed β-subunit (HIF-1β) [23,24,25,26,27]. These subunits share three conserved structural domains critical for their function [28]: (1) N-terminal basic helix–loop–helix (bHLH) domain: Located in the amino-terminal region, this domain facilitates dimerization through its helix–loop–helix (HLH) motif and enables DNA binding via its basic residues, a hallmark of eukaryotic transcription factors. (2) Per-ARNT-Sim (PAS) domain: Positioned centrally, the PAS domain mediates heterodimer assembly through interactions with partner subunits. (3) C-terminal transactivation domain (TAD): The carboxyl terminus houses TADs (TAD-N and TAD-C in HIF-1α), which recruit transcriptional coactivators to drive target gene expression [28]. Furthermore, all HIF-α subunits differ from HIF-1β in that their structures contain an oxygen-dependent degradation domain (ODDD) that overlaps with the N-TAD. This ODDD plays a key role in mediating the regulation of HIF-α stability by oxygen [29].

**Figure 1:**
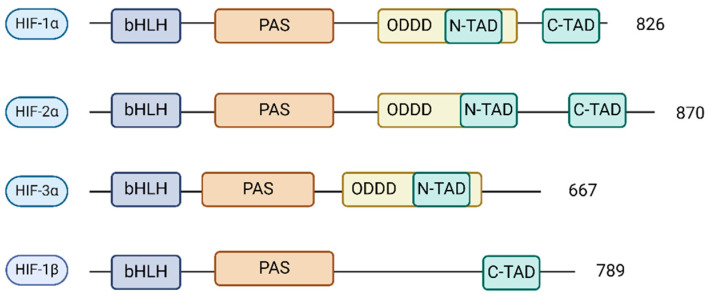
Domain structure of the HIF heterodimer. The HIF family comprises heterodimeric proteins with an oxygen-sensitive α-subunit (HIF-1α, HIF-2α, or HIF-3α) and a constitutively expressed β-subunit (HIF-1β). HIF-1α (826 amino acids) and HIF-1β (789 amino acids) dimerize via conserved N-terminal domains: two Per-ARNT-Sim (PAS-A and PAS-B) subdomains and a HLH motif. A basic region upstream of the HLH domain in both subunits binds HREs in target gene promoters, enabling hypoxia-driven transcriptional regulation. Created with BioRender.com (https://BioRender.com, accessed on 17 March 2026).

Structurally, HIF-1α and HIF-1β comprise 826 and 789 amino acids, respectively. Their heterodimerization depends on conserved regions within the N-terminal half, including the HLH motif and two PAS subdomains (PAS-A and PAS-B) (Figure 1). Sequences upstream of the HLH domain in both subunits further enable binding to HREs in target gene promoters, a prerequisite for hypoxia-driven transcription [23].

Under normoxia, HIF-1α stability is tightly regulated: hydroxylation of specific proline residues allows recognition by the von Hippel–Lindau (VHL) E3 ubiquitin ligase complex, leading to ubiquitination and subsequent proteasomal degradation [25]. Hypoxia inhibits this hydroxylation, drastically reducing ubiquitination and enabling HIF-1α accumulation [30]. Although both HIF-1 and HIF-2 regulate hypoxia-responsive transcription, their oxygen-sensitive α subunits differ in tissue distribution, target-gene preference, and biological function. HIF-1α is more broadly expressed, whereas HIF-2α shows a more restricted and cell-type-specific distribution [24].

Given that HIF-1α is the most ubiquitously expressed isoform and the predominant mediator of systemic hypoxic adaptation, the following sections focus specifically on the role of HIF-1α in T2DM and its complications.

## 4. Role of HIF-1α in Glucose Metabolism Imbalances

### 4.1. Molecular Mechanisms of HIF-1α in Insulin Regulation

DM is fundamentally a metabolic disorder rooted in dysfunctional insulin secretion, impaired insulin action, or both, which disrupts glucose homeostasis and directly drives disease progression [31]. Insulin, the central hormone governing glucose metabolism, regulates peripheral tissue glucose uptake and utilization, with its dysregulation serving as the primary catalyst for hyperglycemia [32]. Hypoxia and immune-metabolic crosstalk have been increasingly recognized as important contributors to the pathogenesis of T2DM [33]. Poor glycemic control not only accelerates diabetic complications such as retinopathy and nephropathy but also establishes a vicious cycle, where these complications further destabilize blood glucose levels, amplifying disease severity [34]. Central to this interplay is hypoxia, which exacerbates insulin resistance and mitochondrial dysfunction across tissues [35].

In cardiomyocytes of diabetic patients, hypoxia and reduced HIF-1α expression impair glucose uptake and exacerbate insulin resistance. Adipose tissue hypoxia activates HIF-1α, shifting cellular metabolism from oxidative phosphorylation to glycolysis—a compensatory mechanism that paradoxically contributes to systemic metabolic dysregulation [6,36]. Mitochondrial reactive oxygen species (ROS) overproduction is a key driver of diabetic complications [37]. Hyperglycemia-induced oxidative stress activates uncoupling protein-2 (UCP-2), which lowers the ATP/ADP ratio and disrupts ATP-dependent processes critical for insulin synthesis, secretion, and signaling. Pancreatic β-cells are particularly vulnerable: oxidative damage from hyperglycemia-generated ROS and reactive nitrogen species (RNS) reduces both insulin production and its biological effectiveness [38]. Furthermore, hyperglycemia-driven mitochondrial hypermetabolism in β-cells alters mitochondrial structure and function, disrupting the function of ATP-sensitive K^+^ channels and thereby impairing glucose-stimulated insulin secretion [38].

Interestingly, HIF-1α overexpression under chronic hyperglycemia reduces mitochondrial ROS accumulation and protects renal epithelial cells from apoptosis [36]. Conversely, adipocyte-specific HIF-1α depletion enhances glucose tolerance by elevating glucagon-like peptide-1 (GLP-1) levels, likely through effects on intestinal L-cells or stability. GLP-1 enhances insulin secretion, promotes β-cell proliferation, and inhibits apoptosis, thereby improving glucose-stimulated insulin secretion and glycemic control [39]. Insulin signaling also regulates adipose regulatory T cell (Treg) plasticity via the HIF-1α-Med23-PPAR-γ axis. In CD73^hi^ST2^lo^ adipose Tregs, deficiencies in insulin receptor, HIF-1α, or Med23 downregulate PPAR-γ, promoting adenosine production and brown adipogenesis [40,41]. Adenosine from these Tregs suppresses inflammation and sustains insulin sensitivity, underscoring their role in metabolic homeostasis [40].

### 4.2. Molecular Mechanisms of HIF-1α in Glucose Transport

Defective glucose uptake, mitochondrial dysfunction, and reduced glycolysis are hallmarks of both type 1 and type 2 diabetes [42]. Glycolysis, the cytoplasmic pathway converting glucose to pyruvate, serves dual roles: generating ATP and directing excess carbon flux toward lipid synthesis, a process critical for energy storage and membrane biosynthesis [43]. Therapeutic strategies targeting these metabolic nodes are being actively investigated. Metformin, a first-line antidiabetic agent, is a widely used insulin sensitizer in current clinical use. Its action regulates multiple biological pathways. With respect to glucose homeostasis, it acts primarily by improving insulin-mediated inhibition of hepatic glucose production and augmenting insulin sensitivity. It ameliorates hyperglycemic symptoms in part by activating AMP-activated protein kinase (AMPK), which enhances glucose transporter-4 (GLUT-4) translocation [44,45]. Insulin orchestrates these metabolic processes by activating glucose transporters GLUT-4 and key glycolytic enzymes, including hexokinase, phosphofructokinase, and pyruvate kinase. Specifically, insulin binds to its receptor, promoting GLUT-4 translocation to the plasma membrane and enhancing glucose influx into cells. Concurrently, insulin stimulates the transcription and post-translational modification of glycolytic enzymes, thereby accelerating glucose catabolism and lowering blood glucose levels [42]. However, in diabetic states, insulin resistance or deficiency disrupts these regulatory mechanisms, leading to hyperglycemia and ectopic lipid accumulation in non-adipose tissues, such as the liver and skeletal muscle.

Under hypoxia, HIF-1α is responsible for reprogramming of metabolic pathways. HIF-1α is essential for many physiological and pathological processes by controlling the expression of genes involved in glucose metabolism, erythropoiesis/iron metabolism, vascular resistance, and circadian rhythms. In most mammalian cells, glucose uptake is mediated by facilitated diffusion through membrane transporters of the GLUT family. HIF-1α acts as a key metabolic regulator and a transcriptional switch. The promoter of GLUT-1 contains HIF-1-binding sites, and HIF-1α directly induces GLUT-1 expression [46,47]. GLUT-1 is ubiquitously expressed in most normal tissues and is responsible for basal glucose transport; under hypoxic conditions, its expression is upregulated to meet increased glycolytic demands [48]. The effects of HIF-1α on glucose metabolism are thus intimately linked to the regulation of glucose uptake, glycolysis, and the tricarboxylic acid (TCA) cycle. Cellular studies demonstrate that HIF-1α critically regulates glucose uptake and glycolytic enzyme activity, significantly amplifying glycolysis [39]. In hypoxia, HIF-1α overexpression drives lactate overproduction and activates signaling pathways that alleviate oxidative stress. HIF-1α upregulates GLUT-1 expression, which is three- to five-fold more abundant under hypoxia, thereby enhancing glucose uptake and glycolysis while suppressing oxidative phosphorylation [39,49,50]. In certain contexts, HIF-1α may also influence GLUT-4 expression or translocation, contributing to increased glucose uptake; GLUT-1 mediates basal uptake, whereas GLUT-4 is the insulin-responsive transporter [51]. These adaptive mechanisms collectively mitigate diabetic progression.

### 4.3. Molecular Mechanisms of HIF-1α in β-Cell Function

T2DM is a chronic metabolic disease defined by persistently elevated blood glucose levels. Pancreatic β-cells, the sole producers of insulin under physiological conditions, are essential for maintaining systemic glucose homeostasis [52]. In healthy β-cells, adaptive functional and qualitative changes effectively counteract insulin resistance to preserve normal glucose tolerance. However, β-cell dysfunction disrupts this equilibrium, progressing to impaired glucose tolerance, elevated fasting glucose, and ultimately overt T2DM [53]. During diabetes development, pancreatic islets undergo compensatory hypermetabolism to offset insulin resistance. This sustained metabolic overload exhausts β-cells, culminating in their failure to regulate blood glucose [21].

β-cells require high oxygen consumption for mitochondrial oxidative phosphorylation to sustain insulin secretion, which renders them highly susceptible to hypoxia. Evidence indicates that hypoxia exacerbates β-cell dysfunction and accelerates T2DM progression [20,21,52]. In cultured rat pancreatic islets, glucose stimulation upregulates glycolytic enzymes and HIF target genes, with this response dependent on both HIF isoforms [20]. Murine studies reveal that β-cell-specific HIF-1α deficiency impairs glucose-stimulated ATP synthesis, reducing insulin production, secretion, and glycolytic gene expression [22,54]. Similarly, mice lacking HIF-1β in β-cells develop glucose intolerance due to defective insulin secretion and altered islet gene profiles [54,55]. Conversely, HIF-1β overexpression in hypoglycemic islets enhances β-cell metabolic signaling, and presenilin upregulation induces HIF-1β expression [55]. These findings further suggest that the HIF-1 heterodimer (comprising HIF-1α and HIF-1β) plays a key role in β-cell metabolic adaptation, and that the initiation and degree of this effect are largely determined by the HIF-1α subunit. In contrast to the established role of HIF-1α in β-cell metabolic adaptation, the contribution of HIF-2α remains less clearly defined. In one β-cell-specific overexpression model, HIF-2α did not substantially alter systemic glucose homeostasis [54], suggesting that the relationship between the two isoforms is not uniformly antagonistic across tissues. Further cell-specific loss- and gain-of-function studies are required to determine whether HIF-2α modifies the narrow HIF-1α activity window in β-cells.

Notably, both HIF-1α deficiency and excessive HIF-1α activation in β-cells have been associated with impaired insulin secretion, indicating that HIF-1α activity must be maintained within an optimal range. This narrow therapeutic window poses a significant challenge for HIF-1α-targeted therapies in diabetes.

## 5. The Role of HIF-1α in Diabetic Complications

### 5.1. Molecular Mechanisms of HIF-1α in Diabetic Kidney Disease (DKD)

Chronic hypoxia is widely recognized as a central pathological mechanism underlying progressive kidney diseases, including diabetic kidney disease (DKD) [56]. As a major microvascular complication of DM, DKD progression involves multiple factors, with disturbances in oxygen and glucose metabolism playing pivotal roles [57]. Among these pathological drivers, dysregulated oxygen metabolism exacerbates renal injury in DKD by promoting hypoxia, oxidative stress, and accumulation of advanced glycation end-products (AGEs) [58,59]. Under diabetic conditions, a complex interplay exists where mitochondrial ROS overproduction can paradoxically influence PHD activity and HIF-1α stability in tubular cells, potentially disrupting adaptive hypoxic responses. Concurrently, AGEs bind to their receptor (RAGE) [60], activating NF-κB and amplifying inflammatory cascades that exacerbate tubular atrophy and interstitial fibrosis [61].

In DKD, HIF-1α activation in renal tubular cells exerts protective effects against renal injury [58,62]. HIF-1α ameliorates mitochondrial dysfunction and limits mitochondria-dependent apoptosis in renal tubular cells through the HO-1 pathway, and HO-1 exerts anti-apoptotic, antioxidant, and anti-inflammatory effects [58] by decreasing the levels of the pro-apoptotic protein Bax [63] and the pro-inflammatory/promotional oxidative protein nitric oxide synthase (iNOS), as well as by increasing the levels of the anti-apoptotic protein Bcl-xl and the anti-nitrosative protein bilirubin [58]. HIF-1α activation induces pyruvate dehydrogenase kinase, thereby inhibiting pyruvate dehydrogenase, reducing pyruvate entry into the TCA cycle, and shifting metabolism from oxidative phosphorylation toward glycolysis [50]. Additionally, HIF-1α inhibits mitochondrial biogenesis and oxidative function while promoting the autophagic removal of damaged mitochondria, a process critical for cellular adaptation to hypoxia and oxidative stress [64].

HIF-2α and HIF-1α share a similar protein structure and have overlapping yet specific target genes, resulting in synergistic or inhibitory effects [65]. A study found that mitochondria in renal tubular epithelial cells in DKD exhibit characteristics of ferroptosis. Maintaining the homeostasis of HIF-1α/HIF-2α is crucial for mitigating ferroptosis. By upregulating HIF-1α and downregulating HIF-2α, fasting blood glucose and proteinuria can be improved, thereby alleviating renal fibrosis and tubular damage [65].

However, in the long term, the role of HIF-1α in DKD is not entirely protective. Emerging evidence suggests that hypoxia in the DKD microenvironment suppresses fatty acid oxidation and promotes lipid synthesis through the HIF-1α pathway, potentially exacerbating lipotoxicity, inflammation, and fibrosis [66]. This contextual duality—protective in some settings but deleterious in others—underscores the need for nuanced, temporally controlled modulation of HIF-1α in DKD therapy.

### 5.2. Molecular Mechanisms of HIF-1α in Diabetic Retinopathy (DR)

Diabetic retinopathy (DR) is one of the most common and severe microvascular complications of diabetes and represents a leading cause of vision loss and blindness [67]. The pathogenesis of DR is mainly characterized by the release of inflammatory and oxidative cytokines and the sensitivity of the neural retina to noxious local microenvironmental stimuli [68], leading to retinal cell loss, vascular dysfunction and neovascularization [69,70]. Hyperglycemia-induced pathological angiogenesis in retinal endothelial cells is central to DR progression. HIF-1α, a key mediator of cellular adaptation that also interacts with ROS signaling, reduces ROS levels by shifting energy production from oxidative phosphorylation to glycolysis [69]. However, hyperglycemia and hypoxia synergistically stabilize HIF-1α, leading to excessive VEGF expression in a spatiotemporally coordinated manner. Elevated VEGF promotes aberrant angiogenesis and increases endothelial permeability, directly linking HIF-1α activation to pathological retinal vascular remodeling [67].

Peroxisome proliferator-activated receptor γ co-activator-1α (PGC-1α) is a downstream target of HIF-1α and a key regulator of energy metabolism. In retinopathy, upregulation of PGC-1α exerts a clear protective effect by inhibiting reactive oxygen species production, inflammation and vascular dysfunction; conversely, the absence of PGC-1α leads to metabolic dysfunction and retinal degeneration [70,71]. However, in hypoxic microenvironments under pathological conditions such as diabetes, activated HIF-1α mediates methylation of the PGC-1α promoter region via EZH2 (enhancer of zeste homologue 2), thereby suppressing PGC-1α expression at the transcriptional level. This negative regulatory relationship has been confirmed by research: following HIF-1α knockdown, PGC-1α expression is significantly up-regulated, indicating that PGC-1α is indeed subject to transcriptional repression by HIF-1α [70]. It is thus evident that the abnormal activation of HIF-1α in disease states suppresses PGC-1α, weakening its endogenous protective function and thereby exacerbating the severity of DR; conversely, inhibiting HIF-1α through therapeutic intervention can lift its transcriptional suppression of PGC-1α, allowing PGC-1α to resume its protective upregulation. These findings strongly support the targeting of HIF-1α as a potential therapeutic strategy for alleviating retinopathy.

### 5.3. Molecular Mechanisms of HIF-1α in Adiposity and Obesity

Obesity and diabetes are two escalating global health challenges, with obesity being a major contributor to T2DM [72]. Obesity, characterized by chronic hypoxia and reduced nitric oxide (NO) bioavailability, triggers low-grade inflammation in adipose tissue, liver, and skeletal muscle [73]. This inflammatory response promotes systemic insulin resistance, hyperinsulinemia, and glucose intolerance, and is causally linked to adipose tissue hypoxia and chronic inflammation—the central driver of metabolic dysregulation in most T2DM patients [74].

With the onset of obesity, adipose tissue becomes hypoxic. Due to uncoupled respiration in adipocytes, obese patients have reduced capillary density and reduced perfusion of adipose tissue, which impairs oxygen delivery and leads to hypoxia [75]. Hypoxia stabilizes HIF-1α in adipocytes, which, alongside NF-κB, amplifies inflammatory pathways and exacerbates metabolic dysfunction. This hypoxic microenvironment recruits classically activated M1 macrophages, perpetuating adipose inflammation [39]. Mechanistically, adipocyte-specific HIF-1α overexpression induces inflammation and insulin resistance by activating fibrotic pathways [75]. HIF-1α upregulates suppressor of cytokine signaling 3 (SOCS3), which inhibits Janus kinase (JAK) activity and subsequently suppresses STAT3-mediated adiponectin production, thereby aggravating insulin resistance [76]. Consistent with these findings, HIF-1α stabilization promotes fibrosis, inhibits adiponectin signaling, and contributes to insulin resistance in experimental obesity models [33]. Adipose tissue inflammation thus serves as a hallmark of obesity and a principal driver of systemic insulin resistance in the context of obesity and T2DM [10,77]. Olefsky and colleagues demonstrated that adipocyte-specific deletion of HIF-1α markedly reduces inflammatory responses in both adipose tissue and liver, while concurrently improving systemic insulin sensitivity in liver and skeletal muscle. In contrast, loss of adipocyte HIF-2α exacerbates inflammation and worsens insulin resistance, suggesting that HIF-2α normally protects against obesity-induced insulin resistance. Further work from this group revealed that HIF-1α actively promotes inflammation and impairs insulin sensitivity, whereas HIF-2α exerts its protective effects, at least in part, by counteracting HIF-1α [10].

Beyond these signaling pathways, HIF-1α also modulates inflammatory responses through the regulation of NO metabolism. Specifically, HIF-1α is capable of upregulating the transcription of inducible iNOS, thereby producing NO, which stimulates inflammation via ROS, impairs adipocyte function, and exacerbates obesity. Mice lacking HIF-1α or HIF-1β in their adipocytes are protected from high-fat diet-induced insulin resistance. Conversely, HIF-2α maintains NO homeostasis by reducing NO production and promoting the expression of arginase 1 (ARG1) [10,78]. To better understand the interaction between the two HIF-α isoforms in obesity, researchers generated adipocyte-specific HIF-1α/HIF-2α double-knockout mice. These double-knockout mice almost exactly replicated all the phenotypes observed in HIF-1α knockout mice: improved glucose tolerance and insulin sensitivity, reduced NO production, and attenuated inflammation and fibrosis in adipose tissue. These results suggest that HIF-1α plays a leading role in promoting the development of obesity-induced insulin resistance, whilst HIF-2α halts the progression of pathological metabolism by inhibiting HIF-1α activity [79]. However, the precise molecular mechanisms underlying their opposing actions in dysfunctional adipocytes remain incompletely understood. Moreover, the possibility that HIF-2α possesses additional protective mechanisms operating in parallel with or independently of HIF-1α should not be excluded and warrants further investigation.

### 5.4. Molecular Mechanisms of HIF-1α in Impaired Wound Healing

With an aging global population and the rising prevalence of diabetes, the burden of diabetic ulcers and impaired wound healing is a growing concern [80]. In diabetic patients, hyperglycemia-induced endothelial dysfunction may impair angiogenesis and further lead to delayed wound healing [80,81]. Impaired wound healing and chronic ulcers in diabetic patients represent a major cause of morbidity and mortality [82,83]. Diabetic wounds, including foot and leg ulcers, affect approximately 20% of people with diabetes [84]. Globally, diabetic wounds are a major health issue. Impaired and delayed healing of such wounds leads to complications, including severe infections and lower limb amputations, which have attracted particular clinical attention [84].

It has been reported that hypoxia plays a key role in the impaired healing of diabetic wounds, as reduced oxygen supply to injured tissues hinders important cellular repair mechanisms [85]. Hyperglycemia is a characteristic of diabetes, and it causes a series of biochemical changes that interfere with the normal wound healing process. One of these changes is a reduction in oxygen supply to the tissue. Hyperglycemia causes problems with the cells lining the blood vessels and damages small blood vessels, thereby reducing blood flow to the wound and affecting oxygen supply [84]. Wound repair, a dynamic and coordinated process involving epithelialization, angiogenesis, granulation tissue formation, and wound contraction, is critically regulated by HIF-1α [86]. HIF-1α drives the expression of angiogenic factors such as VEGF [80], enhances cellular motility [87], and recruits endothelial progenitor cells to injury sites [88,89]. VEGF, in turn, regulates endothelial cells at different stages of angiogenesis. In the inflammatory phase, VEGF increases vascular permeability, affects the expression of endothelial cell selectins and intercellular adhesion molecules, and ultimately promotes leukocyte recruitment to the site of injury. In the proliferative phase, VEGF strongly stimulates the proliferation of endothelial cells. In addition, VEGF was found to regulate endothelial cell migration. In the remodeling phase, VEGF induces the assembly of endothelial cells to promote lumen formation [90,91].

In endothelial cells, HIF-1α further promotes tissue repair by activating stromal cell-derived factor-1 (SDF-1), a chemokine essential for regenerative signaling [88]. Notably, growth differentiation factor 11 (GDF11) accelerates diabetic wound healing by mobilizing endothelial progenitor cells and stimulating neovascularization through the HIF-1α/VEGF/SDF-1 signaling axis [92]. Furthermore, research has shown that HIF-2α and HIF-1α play different roles in wound healing; the absence of HIF-2α in the epidermis can enhance the skin’s antimicrobial activity, reduce the acute inflammatory response and promote wound repair [93].

## 6. Discussion

DM remains a global epidemic, contributing to morbidity and mortality in millions of individuals across developed and developing nations [94,95]. T2DM is characterized by chronic hyperglycemia, metabolic stress, and progressive injury across multiple organs [96]. Hypoxia, oxidative stress, and inflammation interact within these tissues, but the resulting HIF-1α response is not uniform. Instead, its biological consequences depend on the cell type, disease stage, and duration of pathway activation.

**Figure 2:**
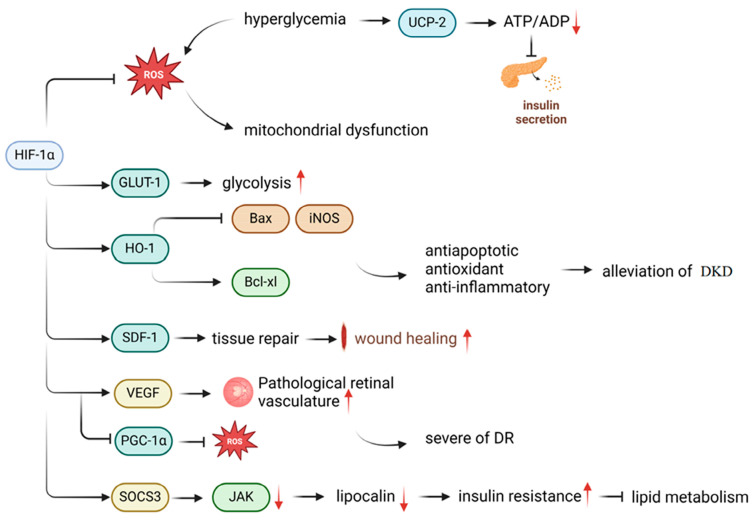
HIF-1α exhibits dual and context-dependent roles in T2DM and its complications. In T2DM, HIF-1α attenuates mitochondrial ROS overproduction while enhancing glucose uptake and glycolysis through upregulation of GLUT-1. In DKD, HIF-1α ameliorates mitochondrial dysfunction in renal tubular cells by inducing HO-1 expression. Concurrently, it promotes wound healing via tissue repair mediated by SDF-1. However, in DR, HIF-1α-driven VEGF overexpression induces pathological neovascularization, contributing to vision loss. Furthermore, in obesity-associated metabolic dysregulation, HIF-1α exacerbates insulin resistance by activating SOCS3. These opposing effects underscore the therapeutic challenge of selectively targeting HIF-1α pathways to balance its protective and detrimental outcomes across diabetic pathologies. Created with BioRender.com (https://BioRender.com, accessed on 17 March 2026).

HIF-1α serves as a core regulator of cellular adaptation to hypoxia and plays a complex and sometimes contradictory role in the development of T2DM and its complications (Figure 2). In fact, the regulation of HIF-1α by hyperglycemia is not inherently contradictory, but rather exhibits cell-context-specificity. In diabetes, although multiple tissues are in a state of hypoxia, due to insufficient activation of the HIF signaling pathway, the stability and function of HIF-1α are frequently suppressed by hyperglycemia and elevated free fatty acids, leading to impaired adaptive hypoxic responses—a phenomenon that has been confirmed in dermal fibroblasts, endothelial cells, cardiomyocytes and proximal tubule cells [97,98,99], with the inhibitory effect of hyperglycemia being dose-dependent and associated with reduced HIF-1α transcriptional activation capacity [6,97]. However, paradoxically, in glomerular mesangial cells, hyperglycemia activates HIF-1α signaling via a mechanism mediated by carbohydrate response element-binding protein (ChREBP) [9]; simultaneously, in adipose tissue of obese individuals, enlarged adipocytes are in a state of relative hypoxia due to insufficient oxygen supply, which is likewise accompanied by the induction of HIF-1α [10]. This seemingly contradictory phenomenon actually stems from the fact that HIF-1α regulation is highly cell-type-dependent and microenvironment-dependent; its ultimate effects depend on the concentration of hyperglycemia, the degree of hypoxia, and the interactions between downstream signaling pathways in different tissues. This differential regulation provides a mechanistic basis for understanding the dual protective and pathogenic roles played by HIF-1α in various diabetic complications.

Beyond tissue-level differences, emerging single-cell and cell-resolved studies reveal substantial heterogeneity in HIF-1α signaling among distinct cell populations within diabetic organs. In DKD, HIF-1α is expressed primarily in the tubular epithelial cells of most nephron segments, whilst HIF-2α is mainly found in vascular endothelial cells and interstitial cells [1,6,7]. In DR, single-cell analysis has shown that HIF-1α is significantly activated in cone cells and retinal Müller cells [8,9]; by upregulating VEGF, it drives pathological angiogenesis, highlighting that even within the same organ, HIF-1α can produce markedly different effects depending on the target cell type. In diabetic wound healing, HIF-1α is significantly up-regulated in fibroblasts, where it activates a variety of downstream factors to promote cell motility, proliferation, angiogenesis, re-epithelialization and cell survival [10]; simultaneously, its stabilization in endothelial cells stimulates VEGF-dependent signaling [11], further enhancing angiogenesis, thereby jointly promoting tissue repair. Furthermore, in DKD, increased stability and expression of HIF-1α in macrophages promote glycolytic reprogramming and are positively correlated with M1 pro-inflammatory macrophage polarization [12], thereby amplifying tissue inflammation. All things considered, these findings shift the understanding of the mechanisms underlying HIF-1α function from a tissue-centred perspective to a cell-type-specific one, and strongly suggest that future therapeutic strategies must not only take the tissue context into account, but also consider the unique HIF-1α regulatory networks within specific cell populations. This is crucial for achieving the precision required for isoform-selective and cell-targeted interventions in the management of diabetic complications.

The HIF-1α signaling pathway plays both a protective and a pathogenic role in T2DM and its complications, making it a potential but challenging therapeutic target. Two broad pharmacological approaches have been investigated. The first involves PHD inhibitors, also referred to as pharmacological HIF stabilizers. By inhibiting PHD-dependent hydroxylation of HIF-α subunits, these agents reduce VHL-mediated proteasomal degradation and increase the abundance of transcriptionally active HIF complexes. Importantly, most PHD inhibitors are not strictly selective for HIF-1α and may also influence HIF-2α signaling. Roxadustat, a representative PHD inhibitor, was approved in China in 2018 for renal anaemia in chronic kidney disease (CKD); clinical trials have demonstrated benefits in dialysis patients, including reduced cardiovascular events and mortality, attenuated endothelial injury, improved vascular stiffness, preserved residual renal function, and enhanced water/sodium excretion [100]. However, its efficacy in diabetic complications remains predominantly supported by preclinical evidence [101,102,103]. The second approach involves inhibition of HIF-1α accumulation or transcriptional activity. Such interventions may reduce HIF-1α-driven pathological angiogenesis, inflammation, or metabolic dysfunction in selected disease contexts. PX-478 has demonstrated efficacy in preclinical models of DR [104] and has also completed a Phase I clinical trial in solid tumours [105]. Although neither Roxadustat nor PX-478 has yet been studied in clinical trials specifically for T2DM and its complications, the accumulated preclinical and clinical data suggest that both classes of agents—and HIF-1α itself—remain promising therapeutic targets.

Despite this promise, however, traditional systemic administration carries the risk of off-target effects and uncontrollable side effects, which limit the clinical translation of these drugs for diabetic complications. In recent years, preclinical advances in tissue-specific delivery systems have opened up new avenues for overcoming this bottleneck: the use of extracellular vesicles (EVs) to deliver the HIF-1α stabilizer VH298 specifically to endothelial cells can selectively enhance angiogenic capacity [80]; co-delivery of HIF-1α stabilizers with alginate-based nanomaterials can simultaneously promote wound hydration and angiogenesis [84]; the newly developed PN@MHV nanocomposite hydrogel utilises folate-modified nanoparticles to target PX-478 to M1 macrophages, thereby achieving bidirectional glycolytic regulation of HIF-1α, which promotes fracture healing in a diabetic model whilst suppressing inflammation [106]. In this context, precision modulation refers to controlling the HIF isoform, target cell type, tissue distribution, disease stage, exposure duration, and dose, rather than uniformly activating or inhibiting the pathway throughout the body. Achieving this goal will require isoform-selective agents, cell-targeted delivery, temporally controlled release, and pharmacodynamic biomarkers that indicate whether HIF-1α signaling is being modulated within the intended therapeutic window. Consequently, future research should focus on integrating the aforementioned small-molecule drugs into advanced nanoscale or hydrogel delivery systems to achieve localised, controlled and on-demand release strategies—thereby fully realising the potential of HIF-1α-targeted therapy whilst minimising systemic exposure, and providing more precise and safer interventions for diabetic complications.

## 7. Conclusions

HIF-1α signaling in T2DM is neither uniformly protective nor uniformly pathogenic. Its effects are determined by the target cell, tissue, disease stage, metabolic environment, and duration of activation. Current evidence supports a narrow functional window in which insufficient HIF-1α impairs adaptation, whereas excessive or persistent activity can promote inflammation, fibrosis, pathological angiogenesis, and metabolic dysfunction. Future studies should combine cell-resolved profiling with isoform-specific perturbation and pharmacodynamic biomarkers to define when, where, and how HIF-1α signaling can be safely modulated.

## Data Availability

No new data were created or analyzed in this study. Data sharing is not applicable to this article.

## Abbreviations

The following abbreviations are used in this manuscript:

DM	diabetes mellitus
HDL	high-density lipoprotein
LDL	low-density lipoprotein
HIFs	hypoxia-inducible factors
HIF-1α	hypoxia-inducible factor 1α
T1DM	type 1 diabetes mellitus
T2DM	type 2 diabetes mellitus
VEGF	vascular endothelial growth factor
EPO	erythropoietin
bHLH	basic helix–loop–helix
PAS	Per-ARNT-Sim
TAD	transactivation domain
HREs	hypoxia response elements
VHL	von Hippel–Lindau
ROS	reactive oxygen species
UCP-2	uncoupling protein-2
RNS	reactive nitrogen species
GLP-1	glucagon-like peptide-1
AMPK	AMP-activated protein kinase
Treg	adipose regulatory T cell
GLUT-1	glucose transporter-1
GLUT-4	glucose transporter-4
DKD	diabetic kidney disease
AGEs	advanced glycation end-products
DR	diabetic retinopathy
PGC-1α	peroxisome proliferator-activated receptor gamma coactivator-1α
NO	nitric oxide
SOCS3	suppressor of cytokine signaling 3
JAK	Janus kinase
iNOS	inducible nitric oxide synthase
ARG1	arginase 1
SDF-1	stromal cell-derived factor-1
GDF11	growth differentiation factor 11
TCA	tricarboxylic acid cycle
EZH2	enhancer of zeste homologue 2
EVs	extracellular vesicles
ChREBP	carbohydrate response element-binding protein
CKD	chronic kidney disease

## References

[1] Pirri D., Fragiadaki M., Evans P.C. (2020). Diabetic atherosclerosis: Is there a role for the hypoxia-inducible factors?. Biosci. Rep..

[2] American Diabetes Association (2009). Diagnosis and Classification of Diabetes Mellitus. Diabetes Care.

[3] Magliano D.J., Boyko E.J. (2021). IDF Diabetes Atlas.

[4] Afsharmanesh M.R., Mohammadi Z., Mansourian A.R., Jafari S.M. (2023). A Review of micro RNAs changes in T2DM in animals and humans. J. Diabetes.

[5] Mansour A., Mousa M., Abdelmannan D., Tay G., Hassoun A., Alsafar H. (2023). Microvascular and macrovascular complications of type 2 diabetes mellitus: Exome wide association analyses. Front. Endocrinol..

[6] Catrina S.-B., Zheng X. (2021). Hypoxia and hypoxia-inducible factors in diabetes and its complications. Diabetologia.

[7] Cowman S.J., Koh M.Y. (2022). Revisiting the HIF switch in the tumor and its immune microenvironment. Trends Cancer.

[8] Chun Y., Kim J. (2021). AMPK–mTOR Signaling and Cellular Adaptations in Hypoxia. Int. J. Mol. Sci..

[9] Isoe T., Makino Y., Mizumoto K., Sakagami H., Fujita Y., Honjo J., Takiyama Y., Itoh H., Haneda M. (2010). High glucose activates HIF-1-mediated signal transduction in glomerular mesangial cells through a carbohydrate response element binding protein. Kidney Int..

[10] Lee Y.S., Kim J.-W., Osborne O., Oh D.Y., Sasik R., Schenk S., Chen A., Chung H., Murphy A., Watkins S.M. (2014). Increased Adipocyte O_2_ Consumption Triggers HIF-1α, Causing Inflammation and Insulin Resistance in Obesity. Cell.

[11] American Diabetes Association (2011). Diagnosis and Classification of Diabetes Mellitus. Diabetes Care.

[12] Darenskaya M.A., Kolesnikova L.I., Kolesnikov S.I. (2021). Oxidative Stress: Pathogenetic Role in Diabetes Mellitus and Its Complications and Therapeutic Approaches to Correction. Bull. Exp. Biol. Med..

[13] Popoviciu M.S., Paduraru L., Nutas R.M., Ujoc A.M., Yahya G., Metwally K., Cavalu S. (2023). Diabetes Mellitus Secondary to Endocrine Diseases: An Update of Diagnostic and Treatment Particularities. Int. J. Mol. Sci..

[14] Fei Z., Xu Y., Zhang G., Liu Y., Li H., Chen L. (2024). Natural products with potential hypoglycemic activity in T2DM: 2019–2023. Phytochemistry.

[15] Zheng Y., Ley S.H., Hu F.B. (2018). Global aetiology and epidemiology of type 2 diabetes mellitus and its complications. Nat. Rev. Endocrinol..

[16] Liu L., Zhang J., Cheng Y., Zhu M., Xiao Z., Ruan G., Wei Y. (2022). Gut microbiota: A new target for T2DM prevention and treatment. Front. Endocrinol..

[17] Sun Y., Tao Q., Wu X., Zhang L., Liu Q., Wang L. (2021). The Utility of Exosomes in Diagnosis and Therapy of Diabetes Mellitus and Associated Complications. Front. Endocrinol..

[18] Nyenwe E.A., Jerkins T.W., Umpierrez G.E., Kitabchi A.E. (2011). Management of type 2 diabetes: Evolving strategies for the treatment of patients with type 2 diabetes. Metabolism.

[19] Sato Y., Endo H., Okuyama H., Takeda T., Iwahashi H., Imagawa A., Yamagata K., Shimomura I., Inoue M. (2011). Cellular Hypoxia of Pancreatic β-Cells Due to High Levels of Oxygen Consumption for Insulin Secretion In Vitro. J. Biol. Chem..

[20] Bensellam M., Duvillié B., Rybachuk G., Laybutt D.R., Magnan C., Guiot Y., Pouysségur J., Jonas J.-C. (2012). Glucose-Induced O_2_ Consumption Activates Hypoxia Inducible Factors 1 and 2 in Rat Insulin-Secreting Pancreatic Beta-Cells. PLoS ONE.

[21] Ilegems E., Bryzgalova G., Correia J., Yesildag B., Berra E., Ruas J.L., Pereira T.S., Berggren P.-O. (2022). HIF-1α inhibitor PX-478 preserves pancreatic β cell function in diabetes. Sci. Transl. Med..

[22] Cheng K., Ho K., Stokes R., Scott C., Lau S.M., Hawthorne W.J., O’Connell P.J., Loudovaris T., Kay T.W., Kulkarni R.N. (2010). Hypoxia-inducible factor-1α regulates β cell function in mouse and human islets. J. Clin. Investig..

[23] Infantino V., Santarsiero A., Convertini P., Todisco S., Iacobazzi V. (2021). Cancer Cell Metabolism in Hypoxia: Role of HIF-1 as Key Regulator and Therapeutic Target. Int. J. Mol. Sci..

[24] Prabhakar N.R., Semenza G.L. (2012). Adaptive and maladaptive cardiorespiratory responses to continuous and intermittent hypoxia mediated by hypoxia-inducible factors 1 and 2. Physiol. Rev..

[25] Yang C., Zhong Z.-F., Wang S.-P., Vong C.-T., Yu B., Wang Y.-T. (2021). HIF-1: Structure, biology and natural modulators. Chin. J. Nat. Med..

[26] Semenza G.L. (2012). Hypoxia-inducible factors: Mediators of cancer progression and targets for cancer therapy. Trends Pharmacol. Sci..

[27] Xin Y., Zhao L., Peng R. (2023). HIF-1 signaling: An emerging mechanism for mitochondrial dynamics. J. Physiol. Biochem..

[28] Liu Y., Xiang D., Zhang H., Yao H., Wang Y., Barreto E. (2020). Hypoxia-Inducible Factor-1: A Potential Target to Treat Acute Lung Injury. Oxidative Med. Cell. Longev..

[29] Masoud G.N., Li W. (2015). HIF-1α pathway: Role, regulation and intervention for cancer therapy. Acta Pharm. Sin. B.

[30] Zhang T., Xu D., Liu J., Wang M., Duan L.-J., Liu M., Meng H., Zhuang Y., Wang H., Wang Y. (2023). Prolonged hypoxia alleviates prolyl hydroxylation-mediated suppression of RIPK1 to promote necroptosis and inflammation. Nat. Cell Biol..

[31] Harreiter J., Roden M. (2019). Diabetes mellitus—Definition, Klassifikation, Diagnose, Screening und Prävention (Update 2019). Wien. Klin. Wochenschr..

[32] Tokarz V.L., MacDonald P.E., Klip A. (2018). The cell biology of systemic insulin function. J. Cell Biol..

[33] Li X., Zhang X., Xia J., Zhang L., Chen B., Lian G., Yun C., Yang J., Yan Y., Wang P. (2021). Macrophage HIF-2α suppresses NLRP3 inflammasome activation and alleviates insulin resistance. Cell Rep..

[34] Atif M., Saleem Q., Babar Z.-U.-D., Scahill S. (2018). Association between the Vicious Cycle of Diabetes-Associated Complications and Glycemic Control among the Elderly: A Systematic Review. Medicina.

[35] Pang H., Luo S., Xiao Y., Xia Y., Li X., Huang G., Xie Z., Zhou Z. (2020). Emerging Roles of Exosomes in T1DM. Front. Immunol..

[36] Fang T., Ma C., Zhang Z., Sun L., Zheng N. (2023). Roxadustat, a HIF-PHD inhibitor with exploitable potential on diabetes-related complications. Front. Pharmacol..

[37] Ridzuan N., John C.M., Sandrasaigaran P., Maqbool M., Liew L.C., Lim J., Ramasamy R. (2016). Preliminary study on overproduction of reactive oxygen species by neutrophils in diabetes mellitus. World J. Diabetes.

[38] Ighodaro O.M. (2018). Molecular pathways associated with oxidative stress in diabetes mellitus. Biomed. Pharmacother..

[39] Gabryelska A., Karuga F.F., Szmyd B., Bialasiewicz P. (2020). HIF-1α as a Mediator of Insulin Resistance, T2DM, and Its Complications: Potential Links with Obstructive Sleep Apnea. Front. Physiol..

[40] Li Y., Lu Y., Lin S.-H., Li N., Han Y., Huang Q., Zhao Y., Xie F., Guo Y., Deng B. (2021). Insulin signaling establishes a developmental trajectory of adipose regulatory T cells. Nat. Immunol..

[41] Cipolletta D., Feuerer M., Li A., Kamei N., Lee J., Shoelson S.E., Benoist C., Mathis D. (2012). PPAR-γ is a major driver of the accumulation and phenotype of adipose tissue Treg cells. Nature.

[42] Holeček M. (2023). Role of Impaired Glycolysis in Perturbations of Amino Acid Metabolism in Diabetes Mellitus. Int. J. Mol. Sci..

[43] Amaral A.I., Hadera M.G., Tavares J.M., Kotter M.R.N., Sonnewald U. (2016). Characterization of glucose-related metabolic pathways in differentiated rat oligodendrocyte lineage cells. Glia.

[44] Herman R., Kravos N.A., Jensterle M., Janež A., Dolžan V. (2022). Metformin and Insulin Resistance: A Review of the Underlying Mechanisms behind Changes in GLUT4-Mediated Glucose Transport. Int. J. Mol. Sci..

[45] Jiao Z., Chen Y., Xie Y., Li Y., Li Z. (2021). Metformin protects against insulin resistance induced by high uric acid in cardiomyocytes via AMPK signalling pathways in vitro and in vivo. J. Cell. Mol. Med..

[46] Oosting M., Kerstholt M., ter Horst R., Li Y., Deelen P., Smeekens S., Jaeger M., Lachmandas E., Vrijmoeth H., Lupse M. (2016). Functional and Genomic Architecture of Borrelia burgdorferi-Induced Cytokine Responses in Humans. Cell Host Microbe.

[47] Xiong S., Liu Z., Yao J., Huang S., Ding X., Yu H., Lin T., Zhang X., Zhao F. (2025). HIF-1α regulated GLUT1-mediated glycolysis enhances Treponema pallidum-induced cytokine responses. Cell Commun. Signal..

[48] Chen C., Pore N., Behrooz A., Ismail-Beigi F., Maity A. (2001). Regulation of glut1 mRNA by Hypoxia-inducible Factor-1: Interaction Between H-ras and Hypoxia. J. Biol. Chem..

[49] Kihira Y., Yamano N., Izawa-Ishizawa Y., Ishizawa K., Ikeda Y., Tsuchiya K., Tamaki T., Tomita S. (2011). Basic fibroblast growth factor regulates glucose metabolism through glucose transporter 1 induced by hypoxia-inducible factor-1α in adipocytes. Int. J. Biochem. Cell Biol..

[50] Kim J.-W., Tchernyshyov I., Semenza G.L., Dang C.V. (2006). HIF-1-mediated expression of pyruvate dehydrogenase kinase: A metabolic switch required for cellular adaptation to hypoxia. Cell Metab..

[51] Siques P., Brito J., Flores K., Ordenes S., Arriaza K., Pena E., León-Velarde F., de Pablo Á.L.L., Gonzalez M.C., Arribas S. (2018). Long-Term Chronic Intermittent Hypobaric Hypoxia Induces Glucose Transporter (GLUT4) Translocation Through AMP-Activated Protein Kinase (AMPK) in the Soleus Muscle in Lean Rats. Front. Physiol..

[52] Wang Y., Liu S., Ying L., Zhang K., Li H., Liang N., Xiao L., Luo G. (2024). Nicotinamide Mononucleotide (NMN) Ameliorates Free Fatty Acid-Induced Pancreatic β-Cell Dysfunction via the NAD+/AMPK/SIRT1/HIF-1α Pathway. Int. J. Mol. Sci..

[53] Eizirik D.L., Pasquali L., Cnop M. (2020). Pancreatic β-cells in type 1 and type 2 diabetes mellitus: Different pathways to failure. Nat. Rev. Endocrinol..

[54] Brunt J.J., Shi S.Y., Schroer S.A., Sivasubramaniyam T., Cai E.P., Woo M. (2014). Overexpression of HIF-2alpha in pancreatic beta cells does not alter glucose homeostasis. Islets.

[55] Dror V., Kalynyak T.B., Bychkivska Y., Frey M.H.Z., Tee M., Jeffrey K.D., Nguyen V., Luciani D.S., Johnson J.D. (2008). Glucose and Endoplasmic Reticulum Calcium Channels Regulate HIF-1β via Presenilin in Pancreatic β-Cells. J. Biol. Chem..

[56] Zheng Q., Zhang X., Guo J., Wang Y., Jiang Y., Li S., Liu Y.N., Liu W.J. (2024). JinChan YiShen TongLuo Formula ameliorate mitochondrial dysfunction and apoptosis in diabetic nephropathy through the HIF-1α-PINK1-Parkin pathway. J. Ethnopharmacol..

[57] Feng X., Wang S., Sun Z., Dong H., Yu H., Huang M., Gao X. (2021). Ferroptosis Enhanced Diabetic Renal Tubular Injury via HIF-1alpha/HO-1 Pathway in db/db Mice. Front. Endocrinol..

[58] Jiang N., Zhao H., Han Y., Li L., Xiong S., Zeng L., Xiao Y., Wei L., Xiong X., Gao P. (2020). HIF-1α ameliorates tubular injury in diabetic nephropathy via HO-1–mediated control of mitochondrial dynamics. Cell Prolif..

[59] Miyata T., Suzuki N., van Ypersele de Strihou C. (2013). Diabetic nephropathy: Are there new and potentially promising therapies targeting oxygen biology?. Kidney Int..

[60] Taneja S., Vetter S.W., Leclerc E. (2021). Hypoxia and the Receptor for Advanced Glycation End Products (RAGE) Signaling in Cancer. Int. J. Mol. Sci..

[61] Tang Y., Wang J., Cai W., Xu J. (2020). RAGE/NF-κB pathway mediates hypoxia-induced insulin resistance in 3T3-L1 adipocytes. Biochem. Biophys. Res. Commun..

[62] Persson P., Palm F. (2017). Hypoxia-inducible factor activation in diabetic kidney disease. Curr. Opin. Nephrol. Hypertens..

[63] Goodman A.I., Olszanecki R., Yang L.M., Quan S., Li M., Omura S., Stec D.E., Abraham N.G. (2007). Heme oxygenase-1 protects against radiocontrast-induced acute kidney injury by regulating anti-apoptotic proteins. Kidney Int..

[64] Packer M. (2021). Mechanisms Leading to Differential Hypoxia-Inducible Factor Signaling in the Diabetic Kidney: Modulation by SGLT2 Inhibitors and Hypoxia Mimetics. Am. J. Kidney Dis..

[65] Yang R., Liu W., Zhou Y., Cheng B., Liu S., Wu R., Liu Y., Li J. (2025). Modulating HIF-1α/HIF-2α homeostasis with Shen-Qi-Huo-Xue formula alleviates tubular ferroptosis and epithelial-mesenchymal transition in diabetic kidney disease. J. Ethnopharmacol..

[66] Yu W., Haoyu Y., Ling Z., Xing H., Pengfei X., Anzhu W., Lili Z., Linhua Z. (2025). Targeting lipid metabolic reprogramming to alleviate diabetic kidney disease: Molecular insights and therapeutic strategies. Front. Immunol..

[67] Ai X., Yu P., Luo L., Sun J., Tao H., Wang X., Meng X. (2022). Berberis dictyophylla F. inhibits angiogenesis and apoptosis of diabetic retinopathy via suppressing HIF-1α/VEGF/DLL-4/Notch-1 pathway. J. Ethnopharmacol..

[68] Loukovaara S., Koivunen P., Inglés M., Escobar J., Vento M., Andersson S. (2014). Elevated protein carbonyl and HIF-1α levels in eyes with proliferative diabetic retinopathy. Acta Ophthalmol..

[69] Sun K., Chen Y., Zheng S., Wan W., Hu K. (2024). Genipin ameliorates diabetic retinopathy via the HIF-1α and AGEs-RAGE pathways. Phytomedicine.

[70] Sun F., Sun Y., Wang X., Zhu J., Chen S., Yu Y., Zhu M., Xu W., Qian H. (2024). Engineered mesenchymal stem cell-derived small extracellular vesicles for diabetic retinopathy therapy through HIF-1α/EZH2/PGC-1α pathway. Bioact. Mater..

[71] Rosales M.A.B., Shu D.Y., Iacovelli J., Saint-Geniez M. (2019). Loss of PGC-1α in RPE induces mesenchymal transition and promotes retinal degeneration. Life Sci. Alliance.

[72] Norouzirad R., González-Muniesa P., Ghasemi A. (2017). Hypoxia in Obesity and Diabetes: Potential Therapeutic Effects of Hyperoxia and Nitrate. Oxidative Med. Cell. Longev..

[73] Piché M.-E., Tchernof A., Després J.-P. (2020). Obesity Phenotypes, Diabetes, and Cardiovascular Diseases. Circ. Res..

[74] Glass C.K., Olefsky J.M. (2012). Inflammation and Lipid Signaling in the Etiology of Insulin Resistance. Cell Metab..

[75] Gonzalez F.J., Xie C., Jiang C. (2018). The role of hypoxia-inducible factors in metabolic diseases. Nat. Rev. Endocrinol..

[76] Jiang C., Kim J.-H., Li F., Qu A., Gavrilova O., Shah Y.M., Gonzalez F.J. (2013). Hypoxia-inducible Factor 1α Regulates a SOCS3-STAT3-Adiponectin Signal Transduction Pathway in Adipocytes. J. Biol. Chem..

[77] Ormazabal V., Nair S., Elfeky O., Aguayo C., Salomon C., Zuñiga F.A. (2018). Association between insulin resistance and the development of cardiovascular disease. Cardiovasc. Diabetol..

[78] Zhou Z., Wang M., Zhang X., Xu Y., Li P., Zheng Z., Yang H. (2025). Synergistic potential of Berberine and Wogonin improved adipose inflammation and insulin resistance associated with obesity through HIF-α axis. Chin. Med..

[79] Kim J., Ban J.-J., Ruthenborg R., Cho K. (2014). Regulation of obesity and insulin resistance by hypoxia-inducible factors. Hypoxia.

[80] Wang Y., Cao Z., Wei Q., Ma K., Hu W., Huang Q., Su J., Li H., Zhang C., Fu X. (2022). VH298-loaded extracellular vesicles released from gelatin methacryloyl hydrogel facilitate diabetic wound healing by HIF-1α-mediated enhancement of angiogenesis. Acta Biomater..

[81] Zhang W., Wang L., Guo H., Chen L., Huang X. (2023). Dapagliflozin-Loaded Exosome Mimetics Facilitate Diabetic Wound Healing by HIF-1α-Mediated Enhancement of Angiogenesis. Adv. Healthc. Mater..

[82] Schmidt A.M. (2018). Highlighting Diabetes Mellitus. Arterioscler. Thromb. Vasc. Biol..

[83] Li G., Ko C.-N., Li D., Yang C., Wang W., Yang G.-J., Di Primo C., Wong V.K.W., Xiang Y., Lin L. (2021). A small molecule HIF-1α stabilizer that accelerates diabetic wound healing. Nat. Commun..

[84] Saber S., Abdelhady R., Elhemely M.A., Elmorsy E.A., Hamad R.S., Abdel-Reheim M.A., El-Kott A.F., AlShehri M.A., Morsy K., Negm S. (2024). Nanoscale Systems for Local Activation of Hypoxia-Inducible Factor-1 Alpha: A New Approach in Diabetic Wound Management. Int. J. Nanomed..

[85] Catrina S.-B., Zheng X. (2016). Disturbed hypoxic responses as a pathogenic mechanism of diabetic foot ulcers. Diabetes/Metab. Res. Rev..

[86] Sunkari V.G., Lind F., Botusan I.R., Kashif A., Liu Z.-J., Ylä-Herttuala S., Brismar K., Velazquez O., Catrina S.-B. (2015). Hyperbaric oxygen therapy activates hypoxia-inducible factor 1 (HIF-1), which contributes to improved wound healing in diabetic mice. Wound Repair Regen..

[87] Hutami I.R., Izawa T., Khurel-Ochir T., Sakamaki T., Iwasa A., Tomita S., Tanaka E. (2022). HIF-1α controls palatal wound healing by regulating macrophage motility via S1P/S1P1 signaling axis. Oral Dis..

[88] Ceradini D.J., Kulkarni A.R., Callaghan M.J., Tepper O.M., Bastidas N., Kleinman M.E., Capla J.M., Galiano R.D., Levine J.P., Gurtner G.C. (2004). Progenitor cell trafficking is regulated by hypoxic gradients through HIF-1 induction of SDF-1. Nat. Med..

[89] Botusan I.R., Sunkari V.G., Savu O., Catrina A.I., Grünler J., Lindberg S., Pereira T., Ylä-Herttuala S., Poellinger L., Brismar K. (2008). Stabilization of HIF-1α is critical to improve wound healing in diabetic mice. Proc. Natl. Acad. Sci. USA.

[90] Huang X., Liang P., Jiang B., Zhang P., Yu W., Duan M., Guo L., Cui X., Huang M., Huang X. (2020). Hyperbaric oxygen potentiates diabetic wound healing by promoting fibroblast cell proliferation and endothelial cell angiogenesis. Life Sci..

[91] Azimi-Nezhad M., Stathopoulou M.G., Bonnefond A., Rancier M., Saleh A., Lamont J., Fitzgerald P., Ndiaye N.C., Visvikis-Siest S. (2013). Associations of vascular endothelial growth factor (VEGF) with adhesion and inflammation molecules in a healthy population. Cytokine.

[92] Zhang Y., Zhang Y.-Y., Pan Z.-W., Li Q.-Q., Sun L.-H., Li X., Gong M.-Y., Yang X.-W., Wang Y.-Y., Li H.-D. (2023). GDF11 promotes wound healing in diabetic mice via stimulating HIF-1α-VEGF/SDF-1α-mediated endothelial progenitor cell mobilization and neovascularization. Acta Pharmacol. Sin..

[93] Cowburn A.S., Alexander L.E.C., Southwood M., Nizet V., Chilvers E.R., Johnson R.S. (2014). Epidermal Deletion of HIF-2α Stimulates Wound Closure. J. Investig. Dermatol..

[94] Wang Y., Beydoun M.A., Min J., Xue H., Kaminsky L.A., Cheskin L.J. (2020). Has the prevalence of overweight, obesity and central obesity levelled off in the United States? Trends, patterns, disparities, and future projections for the obesity epidemic. Int. J. Epidemiol..

[95] Lee M., Hamilton J.A.G., Talekar G.R., Ross A.J., Michael L., Rupji M., Dwivedi B., Raikar S.S., Boss J., Scharer C.D. (2022). Obesity-induced galectin-9 is a therapeutic target in B-cell acute lymphoblastic leukemia. Nat. Commun..

[96] American Diabetes Association (2017). 2. Classification and Diagnosis of Diabetes: Standards of Medical Care in Diabetes—2018. Diabetes Care.

[97] Catrina S.-B., Okamoto K., Pereira T., Brismar K., Poellinger L. (2004). Hyperglycemia Regulates Hypoxia-Inducible Factor-1α Protein Stability and Function. Diabetes.

[98] Marfella R., Esposito K., Nappo F., Siniscalchi M., Sasso F.C., Portoghese M., Pia Di Marino M., Baldi A., Cuzzocrea S., Di Filippo C. (2004). Expression of Angiogenic Factors During Acute Coronary Syndromes in Human Type 2 Diabetes. Diabetes.

[99] Miyata T., Sassa R., Nangaku M., Tanaka T., Inagi R., Fujita T., Ingelfinger J., Katavetin P. (2006). High Glucose Blunts Vascular Endothelial Growth Factor Response to Hypoxia via the Oxidative Stress-Regulated Hypoxia-Inducible Factor/Hypoxia-Responsible Element Pathway. J. Am. Soc. Nephrol..

[100] Yang Y.-T., Wang Y., Qi Y., Fu Z.-H., Hu Y.-P., Wan J.-H., Shi X.-T., Huang J.-Y., He H., Chen Q.-K. (2025). Explore the effect of HIF-PHI on blood pressure variation rate and anemia efficacy in maintenance hemodialysis patients. BMC Nephrol..

[101] Fang T., Ma C., Yang B., Zhao M., Sun L., Zheng N. (2025). Roxadustat improves diabetic myocardial injury by upregulating HIF-1α/UCP2 against oxidative stress. Cardiovasc. Diabetol..

[102] Tang D., Lin Q., Xu K., Lu Q.-P. (2025). Roxadustat: A catalyst for diabetic wound healing through re-epithelialization and angiogenesis. Cytojournal.

[103] Xie R.-Y., Fang X.-L., Zheng X.-B., Lv W.-Z., Li Y.-J., Ibrahim Rage H., He Q.-L., Zhu W.-P., Cui T.-X. (2019). Salidroside and FG-4592 ameliorate high glucose-induced glomerular endothelial cells injury via HIF upregulation. Biomed. Pharmacother..

[104] Wang J., Fang S., Wang J., Liu W., Meng F., Zhou X., Gao L. (2026). Suppression of HIF-1α alleviates the symptoms associated with diabetic retinopathy in mice. Exp. Eye Res..

[105] Lee K., Kim H.M. (2011). A novel approach to cancer therapy using PX-478 as a HIF-1α inhibitor. Arch. Pharmacal Res..

[106] Zhang S., Hu W., Zhao Y., Liao Y., Zha K., Zhang W., Yu C., Liao J., Li H., Zhou W. (2025). Bidirectional modulation of glycolysis using a multifunctional nanocomposite hydrogel promotes bone fracture healing in type 2 diabetes mellitus. Bioact. Mater..

